# Enhanced multifaceted model for plasmon-driven Schottky solar cells with integrated thermal effects

**DOI:** 10.1038/s41598-024-82979-3

**Published:** 2025-01-30

**Authors:** Brahim Aïssa, Ahmer A. Baloch, Adnan Ali, Anirban Mitra

**Affiliations:** 1 Qatar Environment and Energy Research Institute (QEERI), Hamad Bin Khalifa University (HBKU), Qatar Foundation, P.O. Box 34110, Doha, Qatar; 2 Research and Development Center Dubai Electricity and Water Authority (DEWA), Dubai, UAE; 3 Department of Chemical Engineering, Jeju National University, Jeju, 63243 Korea; 4 Department of Physics, Indian Institute of Technology Roorkee, Roorkee, Uttarakhand 247667 India

**Keywords:** Absorption, Plasmon, Schottky, Silicon, Spectral heating, Global analysis, Energy science and technology, Engineering, Materials science, Optics and photonics, Physics

## Abstract

This paper explores the development of an opto-thermal-electrical model for plasmonic Schottky solar cells (PSSCs) using a comprehensive multiphysics approach. We simulated the optical properties, power conversion efficiencies, and energy yield of PSSCs with varying nanoparticle (NP) configurations and sizes. Our spectral analysis focused on the absorption characteristics of these solar cells, examining systems sized 3 × 3, 5 × 5, and 7  × 7, with NP radii ranging from 10 to 150 nm. The study addresses a significant gap in solar cell research by presenting a novel multi-physics energy yield model for PSSCs decorated with gold nanoparticles (Au-NPs) on silicon absorbers. This integrated framework uniquely couples optical, electrical, and thermal responses for the prediction of global energy yield maps. Total spectral heat absorption was evaluated over a range of 300 nm to 1200 nm. This spectral heating was further deconvoluted into nanoparticle heating and thermalization heating in a silicon absorber. The findings indicated that the 5 × 5 NP array with a 70 nm radius enhances electrical performance, with the short-circuit current density (J_sc_) reaching 11.54 mA/cm^2^—A 47% improvement compared to traditional bare silicon Schottky cells of 2 *μ*m thickness. However, this electrical enhancement was also accompanied by a significant increase in heat generation within the nanoparticles, with thermal gains up to 182.5% relative to the bare silicon cells. This substantial rise in thermal energy highlights the critical need for advanced thermal management strategies to mitigate overheating and ensure the overall efficiency of plasmonic-enhanced solar cells. Enhanced energy yield maps confirm the model’s predictions, showing improved outputs globally, especially in sunny regions with potential annual energy yield gains up to 80 kWh/m^2^.

## Introduction

Solar energy stands as a cornerstone in the pursuit of sustainable and renewable energy solutions. Traditional silicon-based photovoltaic (PV) cells have long been the industry standard, but their efficiency and cost remain significant challenges. Recent advancements in nanotechnology, particularly the use of plasmonic nanoparticles (NPs), offer a promising pathway to enhance solar cell performance. Plasmonic Schottky solar cells (PSSCs) combine the beneficial properties of plasmonic NPs with the established efficiency of Schottky junctions, marking a significant leap in photovoltaic technology^1,2^. In the field of plasmon-enhanced solar cells, metal nanoparticles that exhibit local surface plasmon resonance (LSP) are designed to generate highly confined electric fields and significant scattering cross-sections (σ). Nevertheless, these nanoparticles also experience parasitic ohmic losses, which result in localized temperature increases. These thermal effects can negatively impact the photoelectric conversion efficiency and the overall stability of the solar cells^3,4^.

Plasmonic NPs, such as gold (Au) or silver (Ag), can induce strong localized electromagnetic fields when exposed to light, a phenomenon known as surface plasmon resonance (SPR). This effect enhances the light absorption capabilities of the solar cell’s active layer. By concentrating incident light into sub-wavelength volumes, plasmonic NPs increase the optical path length within the absorber material, thus improving overall light absorption. This enhancement allows for thinner absorber layers, achieving similar or higher efficiencies compared to conventional thick layers, which can reduce material costs and improve the economic viability of solar technologies^5–8^. However, thermal effects in plasmon-enhanced Schottky solar cells (PSSCs) should be considered for performance optimization, particularly regarding energy dissipation and stability issues following the incorporation of metal nanostructures^9,10^. Previous studies have demonstrated the potential of plasmonic NPs to enhance the performance of various types of solar cells, including organic, dye-sensitized, and silicon-based cells. Ren et al.^8^ showed that incorporating plasmonic NPs into thin-film solar cells could enhance light trapping and absorption, leading to significant efficiency improvements. Similarly, Sha et al.^9^ highlighted the role of plasmonics in achieving high-efficiency solar cells through enhanced light management. However, much of the existing research has primarily focused on the optical enhancements provided by plasmonic NPs, with less attention given to the accompanying thermal effects. Zhang et al.^10^ emphasized the importance of considering these thermal effects, noting that the heat generated by plasmonic NPs could counteract the benefits of enhanced light absorption if not properly managed.

Despite the promising advantages of plasmonic-enhanced solar cells, there are significant challenges to address. One primary challenge is the thermal effect introduced by plasmonic NPs. While SPR enhances optical absorption, it also generates heat, which can adversely affect solar cell performance by increasing its operating temperature^3,11^. Optimizing NP size, shape, and distribution within the solar cell is crucial for maximizing the benefits of SPR while minimizing adverse thermal effects^11^. Localized surface plasmons (LSPs) in metallic nanoparticles, particularly noble metals like gold and silver, are critical for generating hot electrons through nonradiative decay, a process with significant implications for energy conversion applications such as photodetection, photocatalysis, and photovoltaics^12^.In photovoltaic systems, plasmonic nanoparticles enhance light absorption and increase the efficiency of solar cells by generating hot electrons. When LSPs decay nonradiatively, hot electrons can be injected into the conduction band of nearby semiconductors, such as TiO₂ or ZnO, extending the absorption range to the visible and near-infrared regions. This process enhances light absorption through scattering and increases optical path length, leading to improved photocurrent. In addition to photovoltaics, hot electrons from LSPs play a pivotal role in photocatalysis, especially for water splitting to produce hydrogen^13^. Here, hot electrons generated by plasmonic excitation are injected into the semiconductor conduction band, where they facilitate the reduction of protons to hydrogen, while photoexcited holes drive water oxidation to produce oxygen. This mechanism is promising for solar-to-hydrogen conversion technologies, a critical area of research for sustainable energy. Studies have demonstrated that combining plasmonic nanoparticles, such as gold, with semiconductors like ZnO significantly enhances water-splitting efficiency by improving charge separation and reducing recombination^14^.

For instance, passive radiative cooling, as presented by Fan’s group, has shown promise in cooling down solar cells to enhance photoconversion efficiency^15,16^. This highlights that photovoltaic devices are not solely optical and electrical systems but also inherently thermodynamic systems, with these three physical domains being intricately interconnected^17–19^. Previous simulations of solar cells have predominantly concentrated on optoelectronic analysis, focusing on the electromagnetic and electrical responses to enhance light absorption and optimize carrier collection efficiency^20–23^. However, these studies often assume standard test conditions (STC), neglecting temperature variations that can significantly impact output performance^24–26^. Therefore, investigating electrical, optical, and thermal properties simultaneously, along with accompanying energy dissipation in terms of Ohmic heat loss, is highly desirable for performance optimization^27–29^.

Schottky solar cells leverage the properties of Schottky junctions, which are formed between a metal and a semiconductor. These junctions offer low forward voltage drops and fast switching speeds, making them highly advantageous for solar cell applications. One of the critical benefits of Schottky solar cells is their potential for reduced thickness. Thinner absorber layers can be utilized without sacrificing performance, which not only lowers material costs but also enhances flexibility and reduces the weight of the solar cells. This characteristic is particularly beneficial for applications requiring lightweight and flexible solar panels, such as in portable electronic devices and wearable technology^10^. Plasmonic Schottky solar cells represent a significant advancement by combining the advantageous properties of plasmonic NPs with the efficiency of Schottky junctions. Enhanced light absorption due to plasmonic effects leads to increased carrier generation rates, which are critical for improving the short-circuit current (Jsc) and overall efficiency of the solar cell. Furthermore, the use of plasmonic NPs can potentially reduce the thickness of the silicon absorber layer, making the cells lighter and more flexible, thus expanding their application range.

This study addresses a critical research gap by developing an integrated opto-thermo-electrical model that simultaneously considers the optical, thermal, and electrical aspects of PSSCs. By doing so, it provides a comprehensive understanding of how to optimize these devices for maximum efficiency. The multiphysics framework developed in this study enables the simulation of complex interactions between light, heat, and electrical carriers within the solar cell, providing valuable insights into the design and optimization of PSSCs. Additionally, this study evaluates the global energy yield potential of optimized PSSCs, providing a broader perspective on their applicability and performance across different geographic regions. This analysis is crucial for understanding the real-world impact of plasmonic-enhanced solar cells and their potential to contribute to global renewable energy goals.

## Modeling and simulation details

Our approach involves detailed multiphysics modeling and simulation of PSSCs with various NP configurations. The optical properties are investigated using wave optics simulations to determine the absorption and scattering characteristics of the plasmonic NPs. The electrical performance is evaluated through semiconductor simulations that model carrier transport and recombination processes. Thermal effects are analyzed using heat transfer simulations to assess the temperature distribution within the device and the NPs. Sensitivity analyses are conducted to understand the impact of NP size, array configuration, and fractional coverage on the overall performance of the PSSCs. Plasmonically enhanced configuration is determined by balancing the gains in Jsc with the thermal management of the device. Finally, the global potential of PSSCs is assessed by comparing the annual energy yield of optimized devices with conventional Si Schottky solar cells across different geographic regions.

**Fig. 1:**
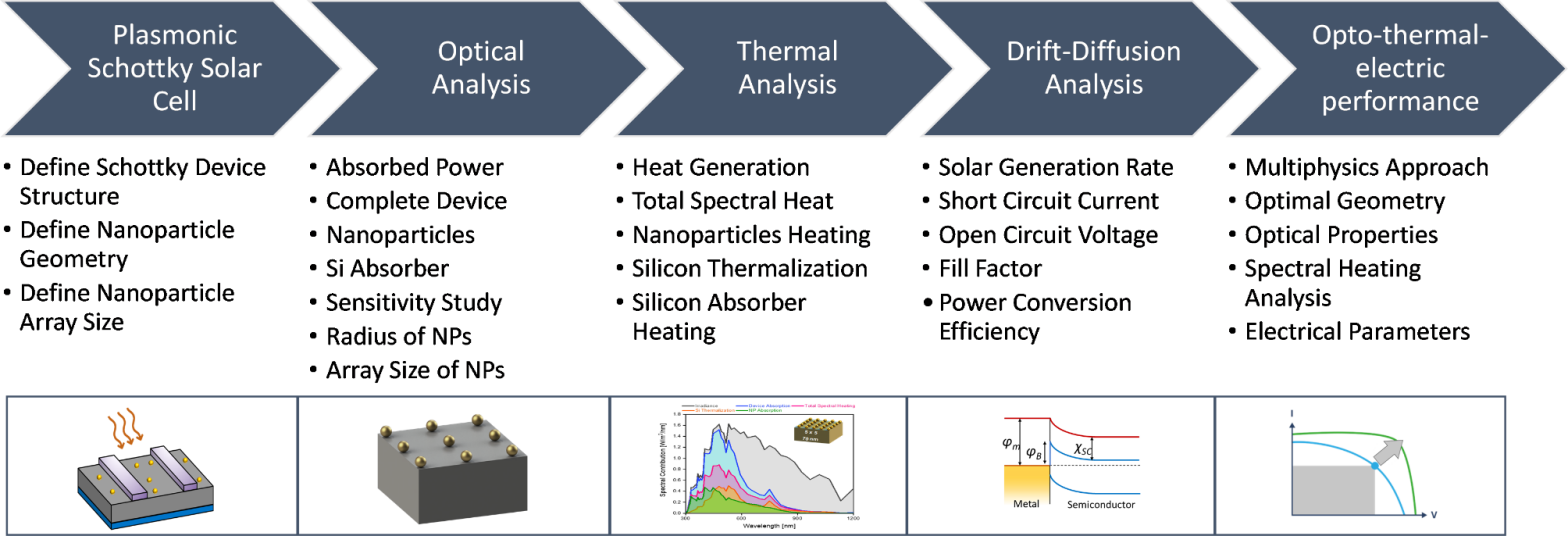
Multi-physics methodology to evaluate the optical, thermal, and electrical characteristics of plasmonic Schottky solar cells.

We have created an opto-thermo-electrical framework using COMSOL for plasmonic Schottky solar cells (PSSCs) with monofacial configuration. These cells are decorated with Au-NPs on the top surface of the silicon (Si) absorber. We investigated the multi-physics effects of Au-NP fractional coverage, examining system size and radius sensitivity. Sensitivity analysis is conducted for system sizes (3 × 3, 5 × 5, 7 × 7) and NP radii (10–150 nm). Figure 1 shows the multi-physics methodology to evaluate the optical, thermal, and electrical characteristics of plasmonic Schottky solar cells. Table 1 shows all the parameters for simulations. The arrays were arranged in 3 × 3, 5 × 5, and 7 × 7 configurations, with NP radii ranging from 10 nm to 150 nm.

**Table 1:** Simulated cases for plasmonic Schottky solar cells analysis. Note: front surface area was kept constant at 3.24 μm^2^.

NP array configuration	NP radius (nm)	Interparticle spacing (nm)
3 × 3	10	870
3 × 3	30	810
3 × 3	50	750
3 × 3	70	690
3 × 3	90	630
3 × 3	110	570
3 × 3	130	510
3 × 3	150	450
5 × 5	10	425
5 × 5	30	375
5 × 5	50	325
5 × 5	70	275
5 × 5	90	225
5 × 5	110	175
7 × 7	10	276.6
7 × 7	30	230
7 × 7	50	183.3
7 × 7	70	136.3
7 × 7	90	90

The multi-physics model was developed by combining modules for wave optics, semiconductor drift-diffusion, and heat transfer. In this study, we optimized the geometric parameters of nanoparticles (NPs) under AM1.5G solar radiation at a temperature of 300 K to enhance light absorption and maximize the short-circuit current (Jsc). To address thermal effects, we employed an energy balance approach to calculate spectral absorption within the device, spectral heating in the NPs, and thermalization heating in the silicon absorber. The combined spectral heating data was then used to determine the temperatures of the device and the NPs. To accurately analyze plasmonic-based solar cells, one must be proficient in three key areas of physical sciences: optics, electrical engineering, and thermal dynamics, as these are essential for precise multi-physics coupling. To achieve this, we utilized various commercial software packages such as COMSOL MULTIPHYSICS^30^, MATLAB^31^, and SCAPS^32^ for the development of plasmonic/solar cell interactions.

Developing plasmonic solar cells (PSC) model is essential in evaluating optoelectronic performance under different geometrical and operational conditions. In essence, PSC modeling represents a multi-physics issue^33,34^ that encompasses both optical and electronic aspects, as depicted in (Fig. 1). Optics pertains to the behavior of plasmonic structures and light propagation, while electronics focuses on solar cell physics, including carrier transport and extraction. Key metrics for optics encompass electric field intensity, resonance modes, absorption (A), reflection (R), and transmission (T). Conversely, the performance of solar cells is determined by carrier generation, transport, recombination, short-circuit current, and quantum efficiency.

Device designs leveraging the propagation of electromagnetic (EM) waves utilize techniques such as surface plasmon polaritons (SPP) in plasmonic waveguides and nanoparticle arrays, as depicted in (Fig. 1b)^35^. Finite difference time domain (FDTD) and discontinuous Galerkin time-domain (DGTD) methods are well-suited for simulating propagation-based plasmonic devices and solving Maxwell’s equations^36^.

### Optical modeling

**Fig. 2:**
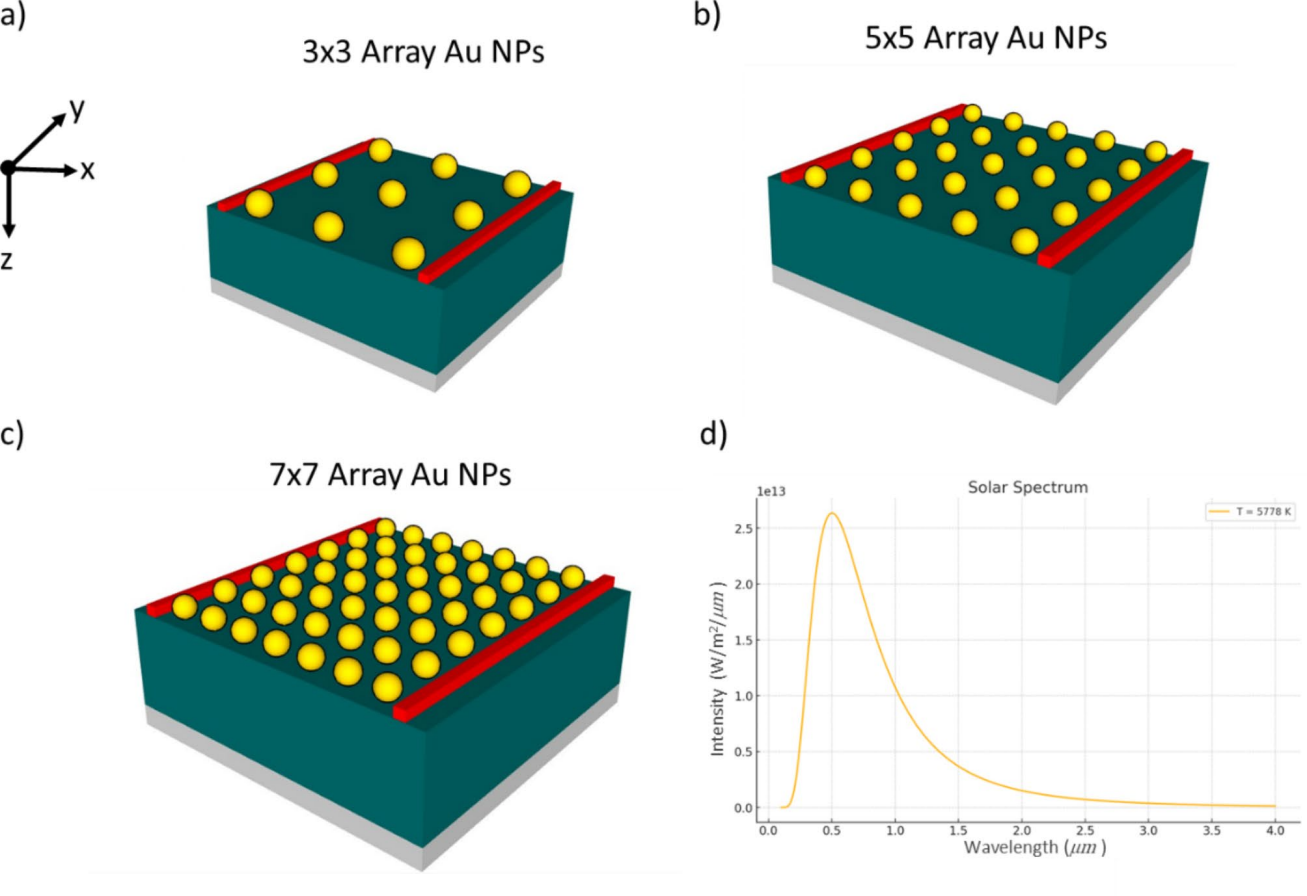
Comparative analysis of plasmonic gold nanoparticle (Au NPs) arrays in solar cells: (**a**) 3 × 3 array, (**b**) 5 × 5 array, (**c**) 7 × 7 array, (**d**) solar spectrum.

We utilized COMSOL to measure optical absorption using the Finite Difference Time Domain (FDTD) method. This approach solves Maxwell’s equations for the electric and magnetic fields within the plasmonic material and the corresponding solar cell geometry. Figure 2 illustrates a comparative analysis of different configurations of gold nanoparticle (Au NPs) arrays used in enhancing the efficiency of solar cells. Figure 2a,b, and 2c display three distinct arrangements of Au NPs: a 3 × 3 array, a 5 × 5 array, and a 7 × 7 array, respectively, each embedded in a substrate. These configurations are studied to determine their effects on the optical properties of the solar cells. Figure 2d shows the solar spectrum with a peak around the visible light range, providing context for the wavelength range that these nanoparticle arrays are optimized to interact with. This setup helps in understanding how varying the density and arrangement of nanoparticles can influence light absorption and overall solar cell performance.

We measure the absorbed power, $$\:{P}_{abs}(x,y,z,\omega\:)$$ as a wavelength function and the generation rate $$\:g(x,y,z,\omega\:)$$ depending on the photon energy and wavelength frequency$$\:\:\left(\omega\:=\raisebox{1ex}{$2\pi\:c$}\!\left/\:\!\raisebox{-1ex}{$\lambda\:$}\right.\right)$$.

$$\:{P}_{abs}\left(x,y,z,\lambda\:\right)=\:\frac{1}{2}\omega\:{\left|E\left(x,y,z,\lambda\:\right)\right|}^{2}\:imag\left\{\epsilon\:\left(x,y,z,\lambda\:\right)\right\}$$

$$\:g\left(x,y,z,\lambda\:\right)=\:\frac{{P}_{abs}\left(x,y,z,\lambda\:\right)}{h\text{c}/\:\lambda\:}$$

The solar spectrum AM1.5G is then used to measure the generation rates to deliver the weighted generation rate as:

$$\:G\left(x,y,z,\lambda\:\right)=g\left(x,y,z,\lambda\:\right)\:\left[\frac{{P}_{solar}\left(\lambda\:\right)}{{P}_{incident}\left(\lambda\:\right)}\right]$$

$\:{P}_{solar}\left(\lambda\:\right)\:$is the AM1.5G incident sun irradiance spectrum and $\:{P}_{incident}\left(\lambda\:\right)$ is the incident power for optical simulations. Lastly, the broadband generation rate is determined by integrating across all wavelengths.

$$\:G\left(x,y,z\right)=\int\:G\left(x,y,z,\lambda\:\right)d\lambda\:$$

Spatially resolved generation profile, $\:G\left(x,y,z\right)$, was then averaged over the volume of the device to yield an effective 1D generation rate $\:{G}_{eff}\left(z\right)$ for use in the SCAPS simulation of an electrical model (next subsection). The formulation for this volumetric averaging is:

$$\:{G}_{eff}\left(z\right)\:=\frac{1}{A}\int\:G\left(x,y,z\right)dx\:dy$$

Where *A* is the cross-sectional area of the device.

### Electrical modeling

For electrical simulations, we employ the diode equations, encompassing Poisson’s equation, drift/diffusion models for electrons and holes, and current-continuity equations. To solve the non-linear Poisson and drift-diffusion equations, we utilize the computational method provided by SCAPS^32^. These equations govern carrier transport and include the Poisson equation, continuity equation, drift-diffusion equation, and recombination currents. Here, $\:{n}_{n,p}$ represents the concentration of electrons/holes, and $\:{G}_{n,p}$ denotes the generation profile derived from an optical simulation.

Poisson Equation:$$\epsilon _{{rel}} \epsilon _{0} \nabla ^{2} \psi = - q~\left( {n_{p} - n_{n} } \right)$$

Continuity:$$\:\:\nabla\:{J}_{n,p}=\left({G}_{n,p}-{R}_{n,p}\left({n}_{p},{n}_{n}\right)\right)$$

Drift-Diffusion:$$\:\:{J}_{n,p}={\mu\:}_{n,p}{n}_{n,p}(-\nabla\:\psi\:)\pm\:{D}_{n,p}\nabla\:{n}_{n,p}$$

Recombination: $$\:{R}_{n,p}\left({n}_{p},{n}_{n}\right)=B\left({n}_{p}{n}_{n}-{n}_{i}^{2}\right)+\frac{{n}_{n}{n}_{p}-{n}_{i}^{2}}{\tau\:({n}_{n}+{n}_{p})}$$

Here, $\epsilon _{{\user2{rel}}}$ is the dielectric permittivity, $\:{\varvec{R}}_{\varvec{n},\varvec{p}}$ is the bimolecular and monomolecular defect-assisted recombination, $\:{\varvec{\mu\:}}_{\varvec{n},\varvec{p}}$ is the electron/ hole mobility, $\:{\varvec{D}}_{\varvec{n},\varvec{p}}$ is the diffusion constant, $\:{\varvec{n}}_{\varvec{i}}$ is the intrinsic carrier concentration, $\:\varvec{B}$ is the recombination coefficient, and $\:\varvec{\tau\:}$ is the lifetime.

The average solar cell photocurrent can be measured as the short circuit current density J_sc_.

$$\:{\text{J}}_{\text{s}\text{c}}\left({\uplambda\:}\right)=\frac{1}{\text{V}\text{o}\text{l}\text{u}\text{m}\text{e}}\underset{0}{\overset{Volume}{\iiint\:}}\left|{\text{J}}_{\text{n}}\left(\text{x},{\uplambda\:}\right)+{\text{J}}_{\text{p}}(\text{x},{\uplambda\:})\right|\text{d}\text{V}\text{o}\text{l}\text{u}\text{m}\text{e}$$

Where V is the solar cell volume, and the integrated short circuit current of the wavelength can be determined as $\:{J}_{sc}=\int\:{J}_{sc}\left({\uplambda\:}\right)\text{d}{\uplambda\:}$.

The response of the solar cell in terms of current-voltage (I-V) characteristics can be described as follows:

$$\:J\left(V\right)={J}_{0}\left[exp\left(\frac{qV}{KT}\right)-1\right]-{J}_{sc}$$

Where J_0_ is the dark saturation reverse current.

The voltage point where the open-circuit voltage (VOC) is observed is when J (V) equals zero:

$$\:{V}_{OC}=\frac{\text{k}\text{T}}{q}ln\left(\frac{{\text{J}}_{\text{s}\text{c}}}{{\text{J}}_{0}}+1\right)$$

Maximum power (P_max_) is:

$$\:{P}_{max}=max\left\{J\left(V\right)\cdot\:V\right\}$$.

Fill factor (FF) is:

$$\:FF=\frac{{P}_{max}}{{J}_{sc}\cdot\:{V}_{OC}}$$

Power conversion efficiency (PCE) is :

$$\:PCE=\frac{{P}_{max}}{{E}_{STC}}$$

Where: $\:{E}_{STC}$ is Incident radiation normalized for the front area.

### Thermal modeling

We computed the anticipated spectral heat content of the plasmonic Schottky junction solar cell devices by utilizing the standard solar spectrum AM1.5 global solar irradiance spectra^37^ denoted as solar ($\:{P}_{solar}$(*λ*)). This calculation involved determining the spectral absorption within the device ($\:{P}_{abs}$ (*λ*)), spectral electrical output ($\:{P}_{elect}$(*λ*)), and the total spectral heat absorbed in the cell ($\:{Q}_{total\:heat\:}$ (*λ*))^38^. Additionally, the spectrum of absorption in nanoparticles (($\:{P}_{abs,NP}$ (*λ*)) and thermalization heating ($\:{P}_{therm,Si}$ (*λ*)) in W/(m^2^.nm) was computed.

The total absorbed power $\:{P}_{abs}$ (*λ*) at any given wavelength λ is calculated by summing the contributions from electrical power $\:{P}_{elect}$(*λ*), power absorbed by nanoparticles $\:{P}_{abs,NP}$ (*λ*) ​, and thermalization power in silicon $\:{P}_{therm,Si}$ (*λ*). This relationship is defined by the equation:

$$\:{P}_{abs}\left(\lambda\:\right)={{P}_{elect}\left({\uplambda\:}\right)+P}_{abs,NP\:}\left(\lambda\:\right){+P}_{therm,Si\:}\left(\lambda\:\right)$$

The electrical power $\:{P}_{elect}\left({\uplambda\:}\right)$ is derived from the product of the maximum power point current $\:{\text{J}}_{\text{m}\text{p}}\left(\lambda\:\right)$and voltage $\:{V}_{mp}\left(\lambda\:\right)$. The power absorbed by nanoparticles$\:{P}_{abs,NP\:}\left(\lambda\:\right)$ is calculated as a fraction of the total absorbed power, $\:{P}_{abs}\left(\lambda\:\right)\times\:{NP}_{abs}\left(\lambda\:\right)$.

The thermalization power in silicon absorber $\:{P}_{therm,Si\:}\left(\lambda\:\right)$ is computed using the excess energy absorbed beyond the bandgap energy Eg, scaled by the incident solar power, as per the formula:

$$\:{P}_{therm,Si\:}\left(\lambda\:\right){=P}_{abs}\left(\lambda\:\right)\times\:\frac{\left({P}_{solar}\left(\lambda\:\right)-{E}_{g}\right)}{{P}_{solar}\left(\lambda\:\right)}$$

The total heat generated $\:{Q}_{\:total\:heat}\left(\lambda\:\right)$ is determined by adding the heat contributions from nanoparticles and the thermalization in silicon for each wavelength. This sum is described by:

$$\:{Q}_{\:total\:heat}\left(\lambda\:\right)={P}_{abs,NP\:}\left(\lambda\:\right){+P}_{therm,Si\:}\left(\lambda\:\right)$$

The absorbed power by nanoparticles, the thermalization power in silicon, and the total heat ​ are integrated over a wavelength range from λ_1_​ to λ_2_​ to provide a comprehensive assessment of the total effect across the solar spectrum.

$$\:{P}_{abs,NP\:}={\int\:}_{{\lambda\:}_{1}}^{{\lambda\:}_{2}}{P}_{abs,NP\:}\left(\lambda\:\right)$$

$$\:{P}_{therm,Si\:}={\int\:}_{{\lambda\:}_{1}}^{{\lambda\:}_{2}}{P}_{therm,Si\:}\left(\lambda\:\right)$$

$$\:{Q}_{total\:heat\:}={\int\:}_{{\lambda\:}_{1}}^{{\lambda\:}_{2}}{Q}_{total\:heat\:}\left(\lambda\:\right)$$

Where Eg​ represents the bandgap of silicon, set at 1.12 eV, and λ1,λ2​ define the boundaries of the integrated heating content, ranging from 300 to 1200 nm, utilized to estimate the heat content of the device.

The device consists of the front surface NP coverage (3 × 3, 5 × 5, and 7 × 7) and a silicon cell absorber. $\:{Q}_{total\:heat\:}$ shows the absorbed radiation for an NP-decorated Si device. It consists of absorption in nanoparticles ($\:{P}_{abs,NP}$) and thermalization heating ($\:{P}_{therm,Si}$). This methodology leverages precise calculations at individual wavelengths to evaluate the overall performance and efficiency enhancements provided by nanoparticle integration in solar cells.

### Energy yield modeling

**Fig. 3:**
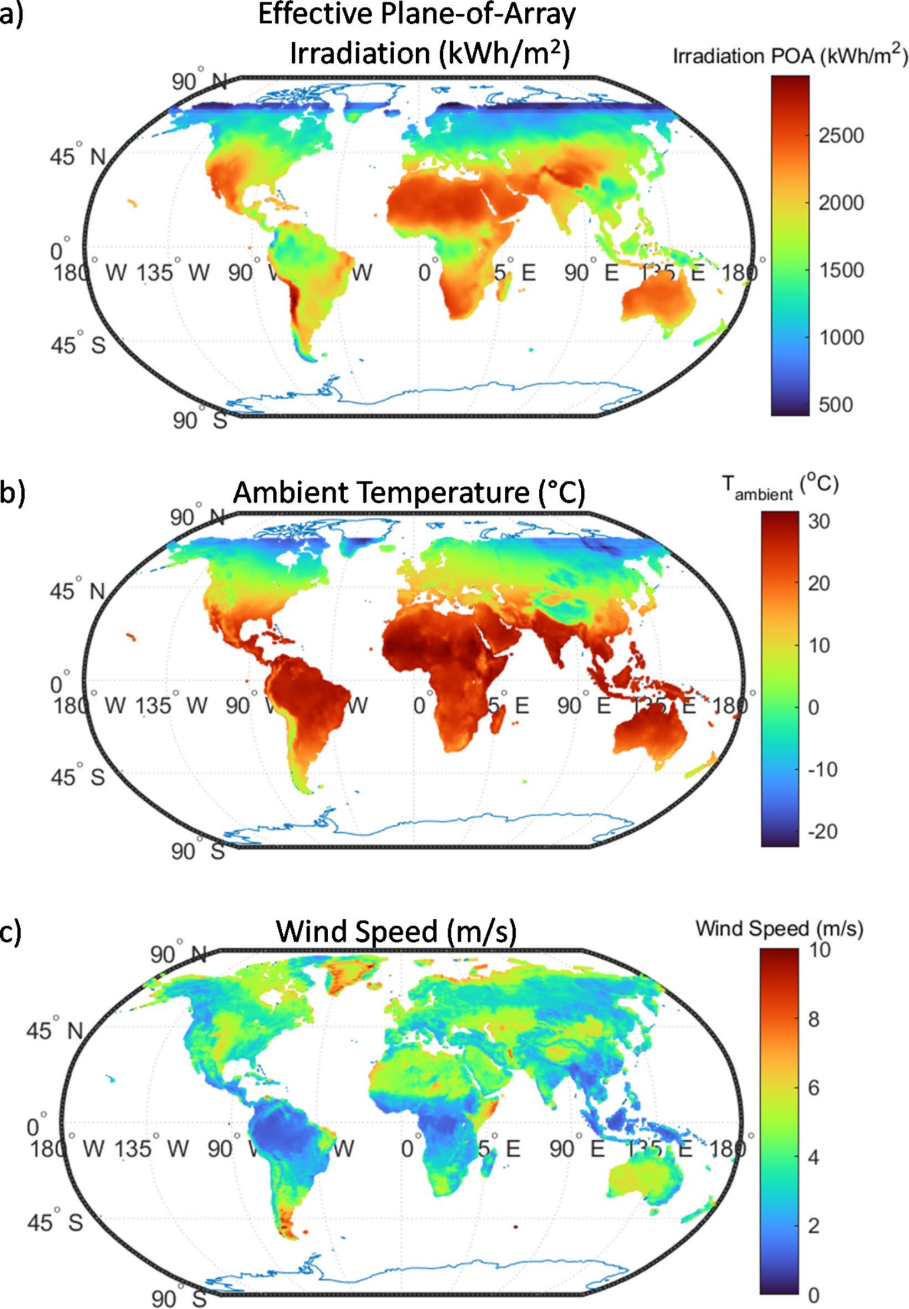
Global average maps of meteorological parameters used in energy yield modeling: (**a**) Effective plane-of-array (POA) irradiation (kWh/m^2^), (**b**) Ambient temperature (°C), and (**c**) Wind speed (m/s).

Following the determination of the ideal nanoparticle (NP) configuration, an analysis of its global real-world potential was conducted using high-resolution meteorological data sourced from satellite observations and relevant studies^39,40^. The annual averaged data, including latitude and longitude, was organized in a fine grid with a resolution of 0.1°, ensuring sufficient spatial granularity to capture local climate variations. Key input parameters for the energy yield model included annual plane of array (POA) incident radiation (kWh/m^2^), wind speed (m/s), and ambient temperature (°C) are shown in (Fig. 3). Ambient temperature data was sourced from gridded historical climate data from 1961 to 1990, collected by the World Meteorological Organization (WMO) stations^39^. This dataset ensured long-term temperature averages were robust, effectively capturing the thermal behavior of the PV modules required for operational energy yield. The annual effective irradiance at the POA, $\:{E}_{eff,POA}$was derived from this gridded dataset, ensuring a comprehensive spatial assessment of system performance across regions. This was obtained from the European centre for medium-range weather forecasts (ECMWF), which relies on dependable ground-based measurements from the baseline surface radiation network (BSRN)^40^. These reliable sources provide accurate inputs for calculating the annual effective irradiance, particularly for modeling performance in varied climatic zones, and can be accessed using References^39,40^. These variables critically influence both the module’s thermal behavior and the amount of incident radiation, impacting the temperature-corrected efficiency and total energy yield of the PV system.

For energy yield calculation, operational efficiency and cell temperature are required. Faiman model was employed to calculate the cell temperature T_c, avg_​, which accounts for the effects of irradiance, ambient temperature, and wind speed^41^. The equation for T_c_ is given by:

$$\:{T}_{c,op}={T}_{amb}+{E}_{eff,POA}\times\:\frac{\left(1-PC{E}_{op}\right)}{{u}_{0}+{u}_{1}\times\:{V}_{w}}$$

- $\:{T}_{amb}$ is the annual average ambient temperature (°C),
- $\:{E}_{eff,POA}$​ is the annual effective irradiance at the plane of the array (kWh/m^2^),
- $\:{T}_{c,op}$​ is the annual average cell temperature (°C),
- PCE is the power conversion efficiency of the cell,
- $\:{u}_{0}$​ and $\:{u}_{1}$​ are heat loss coefficients related to convection and wind cooling (W/m^2^°C),
- V_w_ is the annual average wind speed (m/s).

Following the calculation of T_c, avg_, the cell’s temperature-corrected efficiency, PCE_op_, is determined using the following equation:

$$\:{PCE}_{op}\left({T}_{c,op}\right)=PC{E}_{STC}\times\:\left[1-\gamma\:\left({T}_{c,op}-{T}_{ref}\right)\right]$$

Where:

- $\:{PCE}_{op}\left({T}_{c,op}\right)$ is the temperature-corrected efficiency,
- $\:PC{E}_{STC}$is the cell efficiency at standard testing conditions (AM 1.5 g spectra, 1000 W/m^2^).
- γ is the temperature coefficient of the cell efficiency (%/°C), representing the reduction in efficiency per degree increase in temperature and taken as −0.5%/°C.
- $\:{T}_{ref}$​ is the cell reference standard temperature taken as 25 °C.

Finally, the total annual energy yield $\:E{Y}_{annual}$ ​ is calculated using $\:{PCE}_{op}$ and $\:{E}_{eff,POA}$​ as follows:

$$\:E{Y}_{annual}={E}_{eff,POA}\times\:{PCE}_{op}$$

The linkages between the different modeling aspects of plasmonic solar cells—optical, electrical, thermal, and energy yield—are crucial for accurately simulating overall performance. The optical model directly determines carrier generation rates, which are essential inputs for the electrical model to calculate key parameters such as short-circuit current density (Jsc), open-circuit voltage (Voc), and power conversion efficiency (PCE). Additionally, the optical absorption of photons with energies exceeding the bandgap contributes to heat generation through thermalization, as captured by the thermal model, leading to increased cell temperature. This heat generation adversely impacts electrical performance, as higher cell temperatures reduce PCE due to increased recombination rates and reduced carrier mobility. Furthermore, the energy yield model accounts for these thermal effects, with elevated temperatures negatively impacting the temperature-corrected efficiency, thereby lowering the overall energy yield, especially in hot climates. This integrated approach ensures that optical absorption, carrier transport, thermal effects, and real-world environmental conditions are comprehensively addressed to predict the efficiency and annual energy yield of plasmonic solar cells with high accuracy.

## Results and discussion

**Fig. 4:**
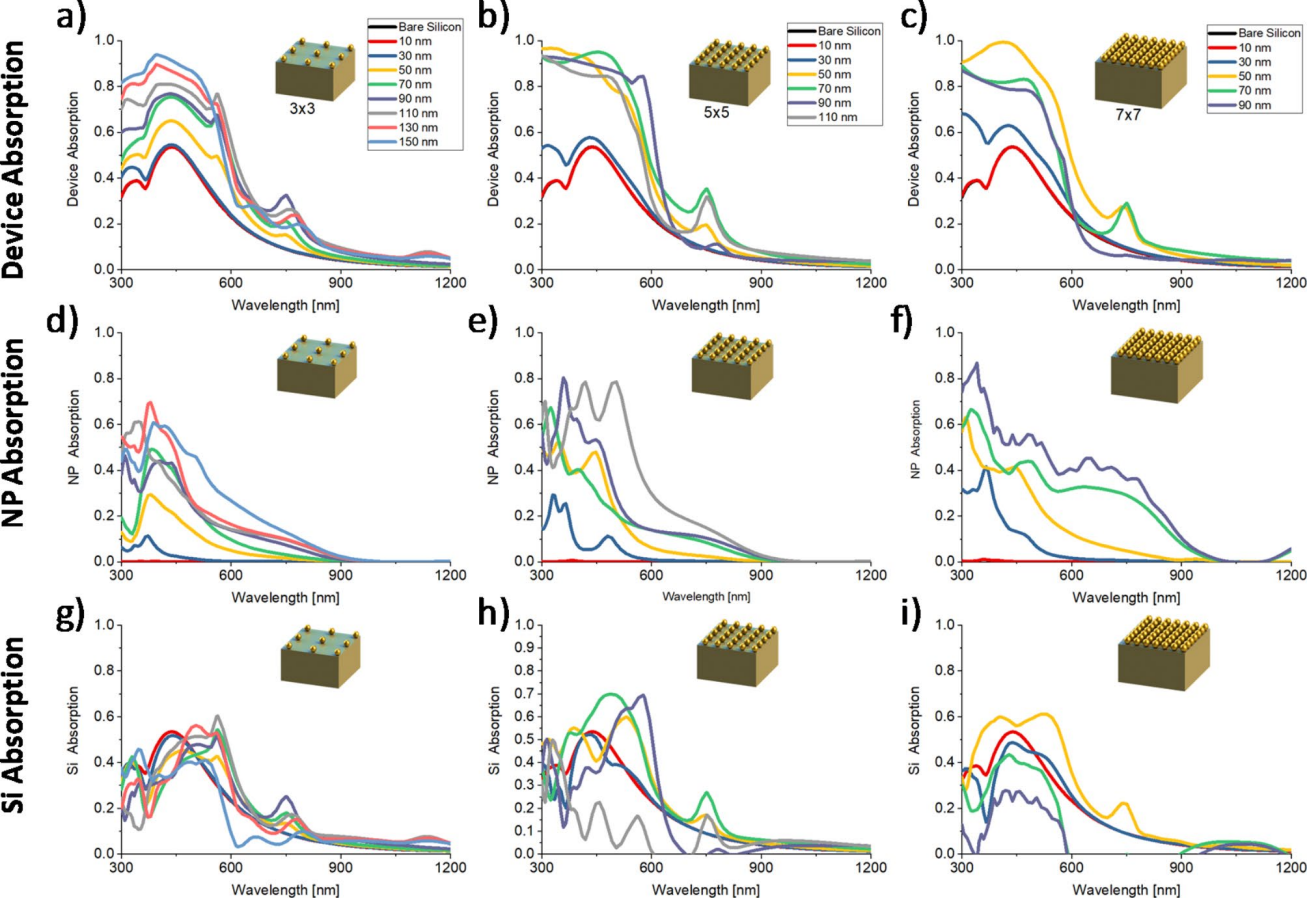
Absorption spectrum for variable systems sizes (3 × 3, 5 × 5, 7 × 7) and NP radii. . (**a**–**c**) Device absorption which can be further segregated into (**d**–**f**) total NP absorption and (**g**–**i**) Si absorber absorption for carrier generation.

Design and analysis of plasmonic Schottky solar cells (PSSC) were undertaken through a comprehensive multiphysics methodology utilizing COMSOL, MATLAB, and SCAPS. This integrated model facilitates the evaluation of optical characteristics, particularly absorption across different layered media within the PSSC, as demonstrated by the spectral absorption curves shown in (Fig. 4). The absorption spectrum of plasmonic Schottky solar cells (PSSC) significantly influences their performance, particularly as it relates to system size and the radii of embedded nanoparticles (NPs). Figure 4 systematically presents these variations, capturing the complicated dynamics between the plasmonic effects induced by NPs and the overall solar cell efficiency. These curves illustrate the absorption spectrum across various configurations, including different system sizes (3 × 3, 5 × 5, 7 × 7) and nanoparticle (NP) radii. In essence, the device’s absorption can be categorized into total nanoparticle absorption and silicon (Si) absorber absorption, which is crucial for carrier generation. The figure is segmented into several panels, each focusing on different aspects of absorption within the cell. Panels ‘a’ through ‘c’ provide a broad overview of different absorption mechanisms. Figure 4a–c highlight a notable increase in absorption within the visible spectrum, which is optimally aligned with the spectral response characteristics of silicon-based solar cells. This enhanced absorption is facilitated by the strategic incorporation of nanoparticle plasmon arrays. This improvement significantly augments the carrier generation potential within the absorber layer, thereby enhancing the overall efficiency of the solar cell. This approach not only maximizes the inherent photovoltaic properties of the silicon but also harnesses the unique benefits of plasmonic phenomena to push the boundaries of solar cell performance. However, panels ‘d’ through ‘f’ explain, illustrating the specific contribution of total NP absorption, and panels ‘*g’* through ‘*i*’ highlight the absorption within the silicon absorber, crucial for carrier generation. This segregation is crucial as it highlights the role of nanoparticles in enhancing light absorption by utilizing plasmonic resonances but at the same time causes heating.

**Fig. 5:**
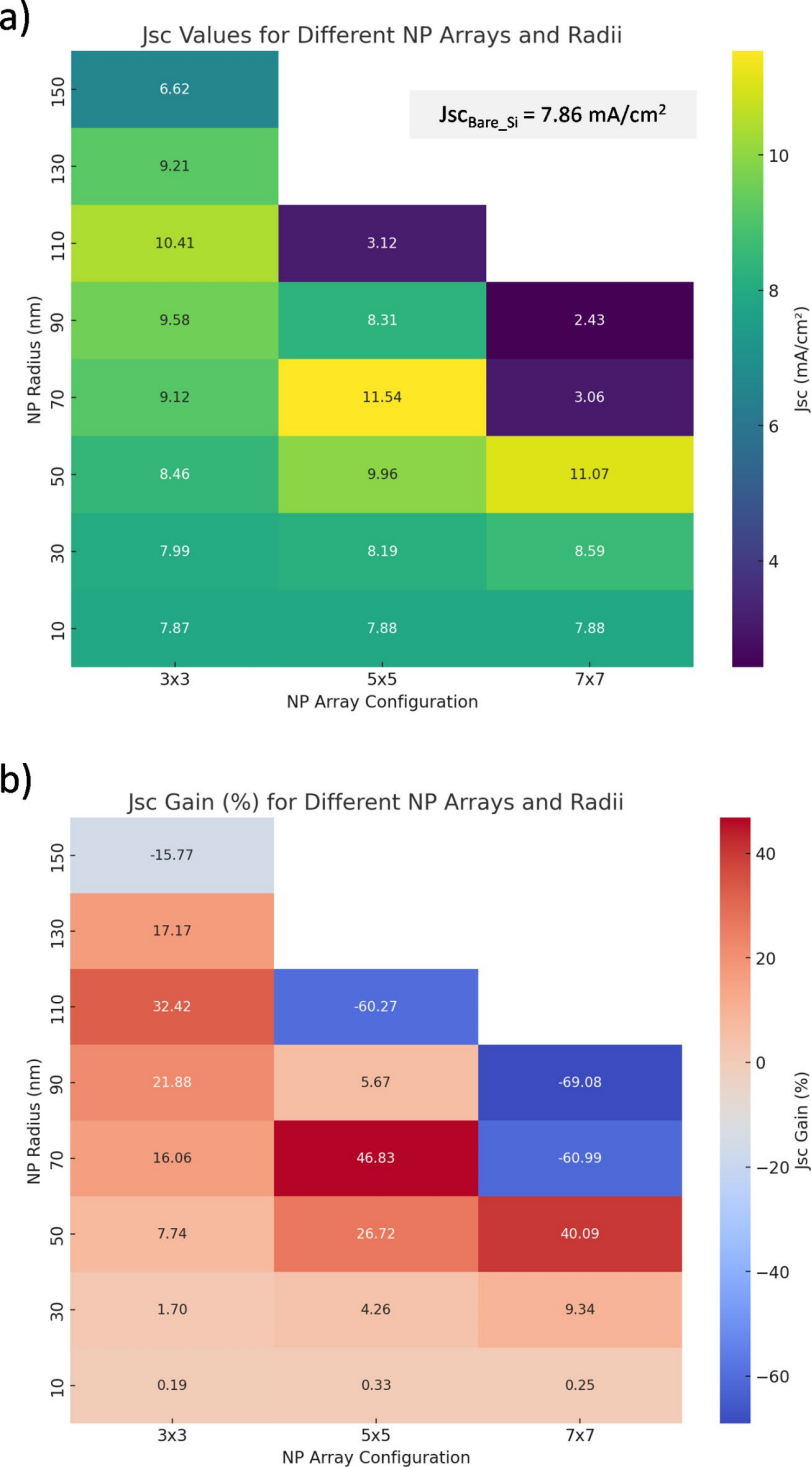
(**a**) Short circuit current (J_*sc*_) generated in Si absorber with Au NP as a function of NP radii and array size. The inset shows a bare silicon Schottky solar device with Jsc_*BareSi*_= 7.86 mA/cm^2^. The color coding serves as an indicator of J_*sc*_ magnitude, with lighter colors denoting higher currents, and (**b**) Short circuit current (J_*sc*_) gain percentage compared to Bare-Si Schottky solar cell. The color gradient transitions from blue, indicating a negative gain, to red signifying a positive gain. Figure created by Python 3.11.7, Dec. 2023, https://www.python.org.

Figure 5 offers a comprehensive visual analysis of the enhancements in plasmonic Schottky solar cells (PSSC) due to the incorporation of gold nanoparticles (Au NPs). This figure is divided into two main panels, each elucidating the impact of nanoparticle arrays and their radii on solar cell efficiency. Figure 5a presents the short-circuit current (Jsc) values measured in milliamperes per square centimeter (mA/cm²) for different NP array configurations (3 × 3, 5 × 5, 7 × 7) and varying radii (10 nm to 150 nm). The color coding serves as an indicator of Jsc magnitude, with lighter colors denoting higher currents. A notable observation is the high Jsc value of 11.54 mA/cm² for the 70 nm radius NPs in a 5 × 5 array, suggesting that this configuration effectively balances nanoparticle-induced plasmonic resonance with the physical limitations of the cell structure. For instance, at an NP radius of 110 nm, the Jsc for the 3 × 3 array reaches a maximum 10.4 mA/cm^2^, while the 5 × 5 configuration shows a notable enhancement with a value of 11.54 mA/cm^2^ at 70 nm NP. This demonstrates how plasmonically enhanced size and array size can harness localized surface plasmon effects to improve light absorption.

The second panel Fig. 5b compares the percentage gain in Jsc when utilizing Au NPs against a baseline of a bare silicon (Bare-Si) Schottky solar cell. The color gradient transitions from blue, indicating a negative gain, to red, signifying a positive gain. This gradient illustrates the varied effects of different configurations; for instance, the 5 × 5 array with 70 nm radius NPs shows a significant increase in Jsc by 46.83%, highlighting its exceptional performance enhancement. Interestingly at 130 nm, the 3 × 3 configuration exhibits a gain of approximately 17.17% relative to bare silicon, while at 150 nm, a decrease of about 15.77% is observed, indicating potential drawbacks of larger NPs after the optimal stage due to increased lossy absorption and shielding effects.

In contrast, smaller NPs in larger arrays, like the 7 × 7 array with 10 nm NPs, occasionally exhibit a decrease in performance, showcasing the intricate balance between NP size, array configuration, and overall photovoltaic efficiency. Jsc negative gain values for smaller NPs indicate that they may not effectively scatter light, while larger NPs can introduce complex scattering dynamics that affect overall absorption profiles. The spectral response suggests that as the NP radius increases, the scattering can initially enhance light absorption until reaching a critical size, beyond which the efficiency declines due to increased losses.

In the context of plasmonic solar cells, the spacing between metallic nanoparticles (NPs) is a critical factor that influences light absorption and overall device performance. Given the constant simulation area of 1.8 μm by 1.8 μm, the arrangement and spacing of NPs must be strategically considered to optimize their impact on the short-circuit current density (Jsc). For the simulation of nanoparticle (NP) arrays, we used a fixed front area of 1.8 by 1.8 micrometers squared for incident radiation. Within this area, we computed the interparticle spacing for different NP configurations and radii. Table 1 shows all the parameters for simulations. The arrays were arranged in 3 × 3, 5 × 5, and 7 × 7 configurations, with NP radii ranging from 10 nm to 150 nm. For example, in the 3 × 3 configuration with a 10 nm radius, the interparticle spacing was calculated to be 520 nm. As the NP radius increased, the interparticle spacing decreased, such as in the 3 × 3 configuration with a 50 nm radius, where the spacing was 400 nm. This trend holds for all configurations, as larger particles occupy more of the total area, reducing the spacing between them. The method ensured equal particle spacing within the fixed simulation area and allowed us to systematically examine the effects of NP size and array configuration on the optical and thermal properties of the system.

Regarding the impact of interparticle spacing, the spacing between AuNPs is critical for influencing the overall optical response of the system. Smaller interparticle gaps result in stronger plasmonic coupling, leading to higher local field intensities and improved light absorption. However, closer proximity can also lead to increased scattering or heating effects. Larger spacing, on the other hand, can reduce the plasmonic coupling between particles, diminishing the enhancement in light absorption. Hence, the spacing has a significant impact on the performance of plasmonic solar cells. The Si substrate considered in the simulations had a fixed area of 1.8 by 1.8 micrometers squared. For different substrate sizes, the design of the AuNP arrays should be adapted to maintain optimal interparticle spacing. For larger substrate areas, the number of nanoparticles and the spacing between them should be adjusted to ensure effective plasmonic enhancement and light absorption, without introducing excessive scattering or thermal losses. Maintaining appropriate spacing in larger arrays would be essential to preserving the plasmonic effects that improve the optical and electrical performance of the system. All of these effects have been further shown in (Figs. 4 and 5).

**Fig. 6:**
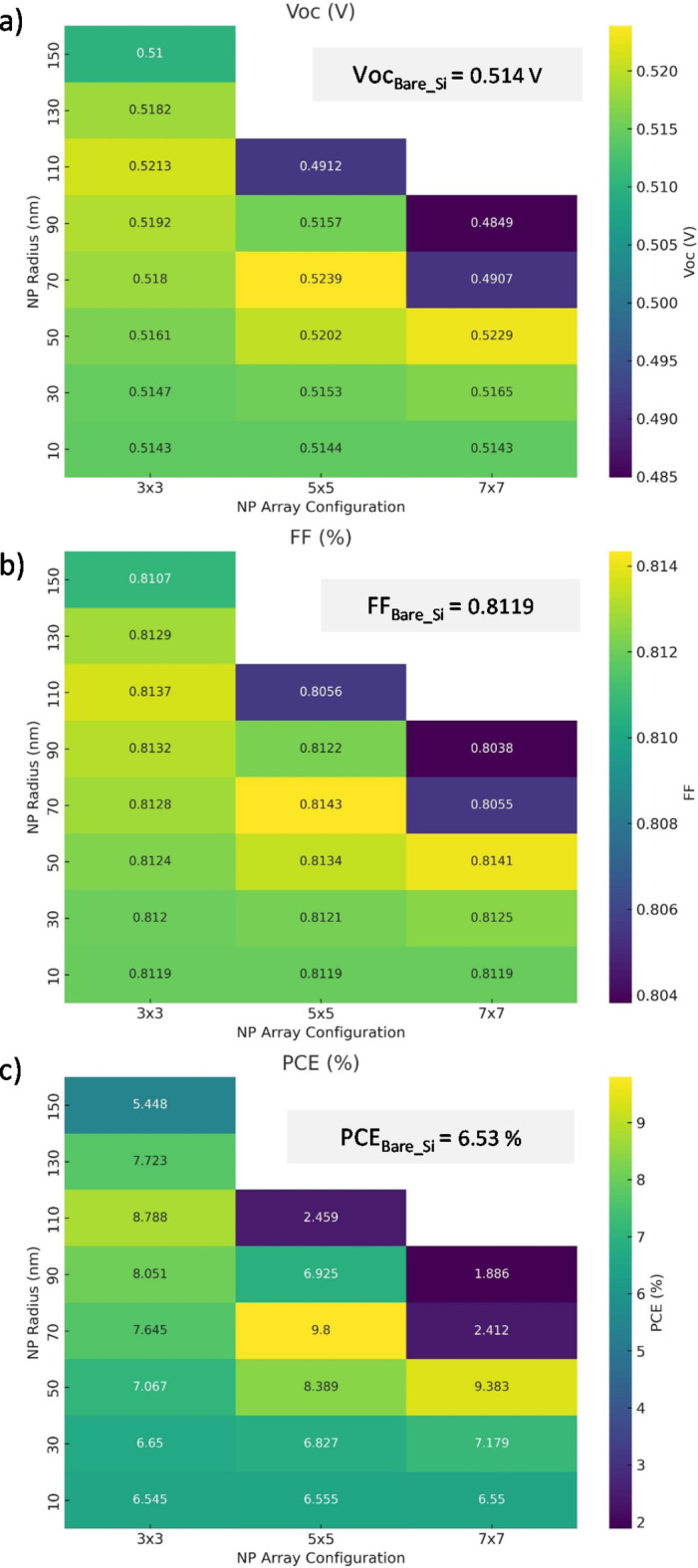
Heatmaps showing the relationship between NP radius and various photovoltaic parameters: (**a**) Open circuit voltage (Voc), (**b**) Fill factor (FF), and (**c**) Power conversion efficiency (PCE) across different NP array configurations (3 × 3, 5 × 5, and 7 × 7). Figure created by Python 3.11.7, Oct. 2024, https://www.python.org.

Figure 6 shows that incorporating plasmonic nanoparticles into silicon solar cells significantly alters their electrical performance, particularly in terms of power conversion efficiency (PCE)- due to large variations explained earlier in Jsc. The integration of plasmonic nanoparticles (NPs) into silicon solar cells consistently enhances performance, particularly in terms of power conversion efficiency (PCE), open-circuit voltage (Voc), and fill factor (FF), though the extent of these improvements depends heavily on NP size and array configuration. Figure 6a demonstrates that adding NPs generally increases Voc, as seen in the 3 × 3 configuration, where Voc rises from 0.514 V for a 10 nm NP radius to 0.521 V for a 110 nm radius. Similar trends are observed in the 5 × 5 and 7 × 7 arrays, with the 5 × 5 configuration achieving the highest Voc of 0.524 V at a 70 nm NP radius. This suggests that NPs enhance light absorption and charge carrier generation. Figure 6b shows that FF remains relatively stable around 81% across most configurations for smaller NP radii, but begins to decline slightly with larger NP sizes, especially in the 7 × 7 array, where a 90 nm NP radius reduces the FF to 80.38%. This indicates that larger NPs may introduce additional recombination losses, slightly hindering charge extraction. The most notable improvements are observed in PCE, with substantial gains seen in both the 3 × 3 and 5 × 5 configurations – primarily coming from Jsc enhancement as depicted in Fig. 6c. In the 3 × 3 array, PCE increases from 6.55% at a 10 nm NP radius to a maximum of 8.79% at a 110 nm radius. The 5 × 5 configuration exhibits the highest PCE, peaking at 9.80% for a 70 nm NP radius—significantly outperforming bare silicon’s PCE of 6.53%. In the 7 × 7 configuration, however, the PCE reaches a maximum of 9.38% at a 50 nm NP radius, but declines sharply for larger NP sizes, dropping to 1.89% for a 90 nm NP radius. These trends highlight that while plasmonic NPs can significantly improve efficiency through enhanced light trapping, oversized NPs may lead to detrimental effects such as increased scattering or recombination, reducing overall performance. When compared to bare silicon, which has a PCE of 6.53%, FF of 81.19%, and Voc of 0.514 V, the inclusion of optimally sized NPs, particularly in the 5 × 5 and 3 × 3 arrays with NP radii of 50–70 nm, offers efficiency improvements of up to 50%. However, poorly tuned NP sizes—especially in the 7 × 7 array with larger NPs—can result in efficiency losses, demonstrating the importance of precise tuning.

**Fig. 7:**
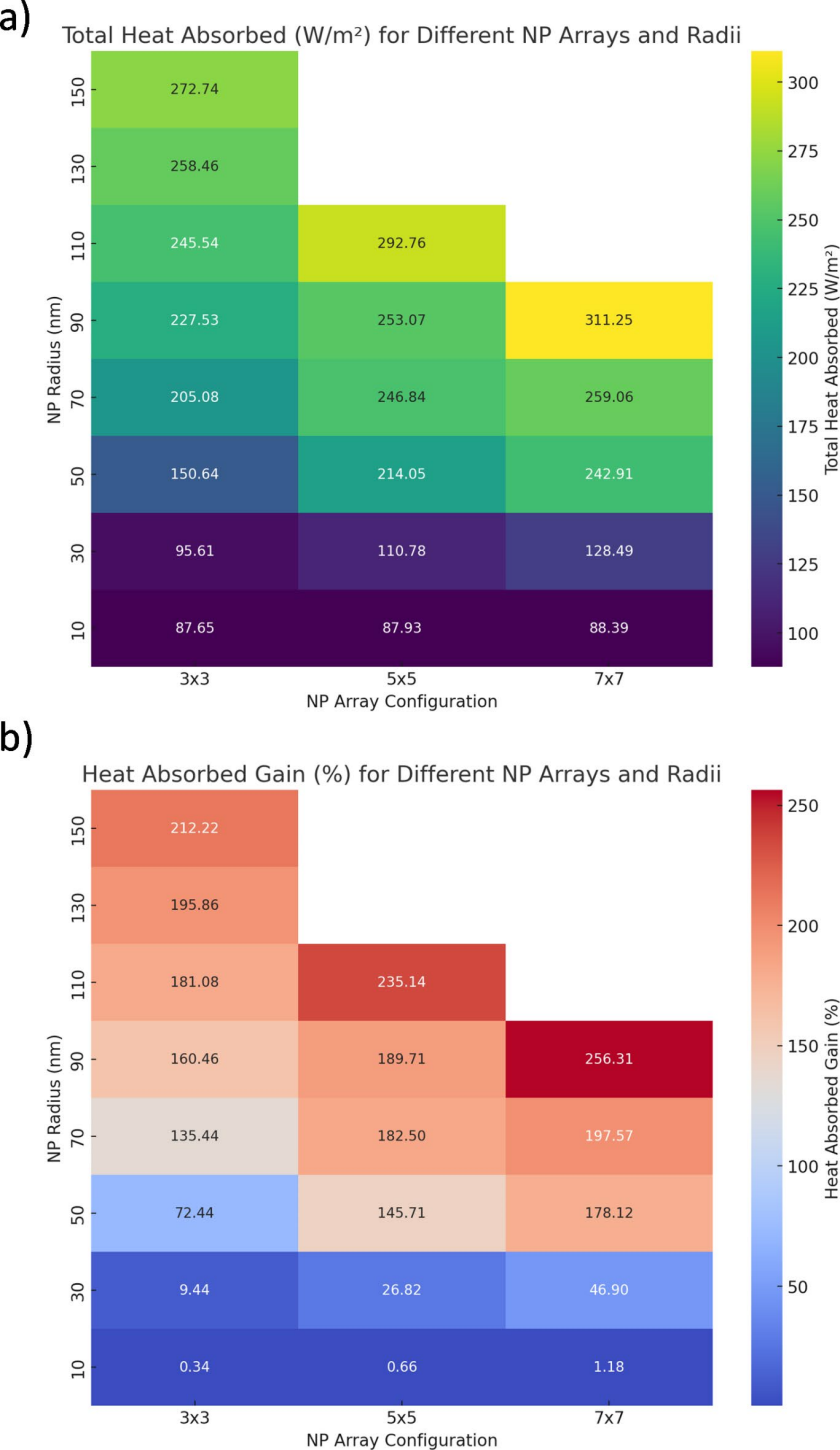
(**a**) Total spectral heat absorbed in PSSC as a function of NP radii and array size and (**b**) Total spectral heat gain (%) compared to Bare-Si Schottky solar cell. Figure created by Python 3.11.7, Dec. 2023, https://www.python.org.

Figure 7 shows the total spectral heat absorbed in PSSC as a function of NP radii and array size and the total spectral heat gain (%) when compared to bare-Si Schottky solar cells. Total spectral heat absorbed,$\:\:{Q}_{total\:heat\:},$ is obtained by integrating total spectral heating from 300 nm to 1200 nm. Generally, a higher fraction of NP array and NP radius were found to increase the total spectral heating content as shown in (Fig. 7a). The highest heating absorption is noted at 311.25 W/m² for a 7 × 7 array with a nanoparticle radius of 110 nm. This is primarily due to the additional plasmonic absorption by NPs.

For a plasmonic enhanced configuration from a solar cell perspective, i.e. less heating, $\:{Q}_{total\:heat\:}$was found to be 246.83 W/m^2^ for an array of 70 nm NPs with 5 × 5. These values were then compared to bare-Si Schottky solar cells without NPs as shown in (Fig. 7b). It shows that larger heat gains are predominantly observed with larger nanoparticle radii and denser configurations. When compared with bare cells, the gain in $\:{Q}_{total\:heat\:}$was found to be 182.56% for selected plasmonically enhanced case. In Fig. 7a, it’s evident that heat absorption tends to increase with both the array density and the size of the nanoparticles. On the maximum side, a 7 × 7 array with a 150 nm radius exhibits a significant gain of 256.31%, illustrating the highest heat content. This also underscores that while smaller arrays and nanoparticle sizes show gains, these are modest in comparison, as seen with the 3 × 3 array across all radii.

**Fig. 8:**
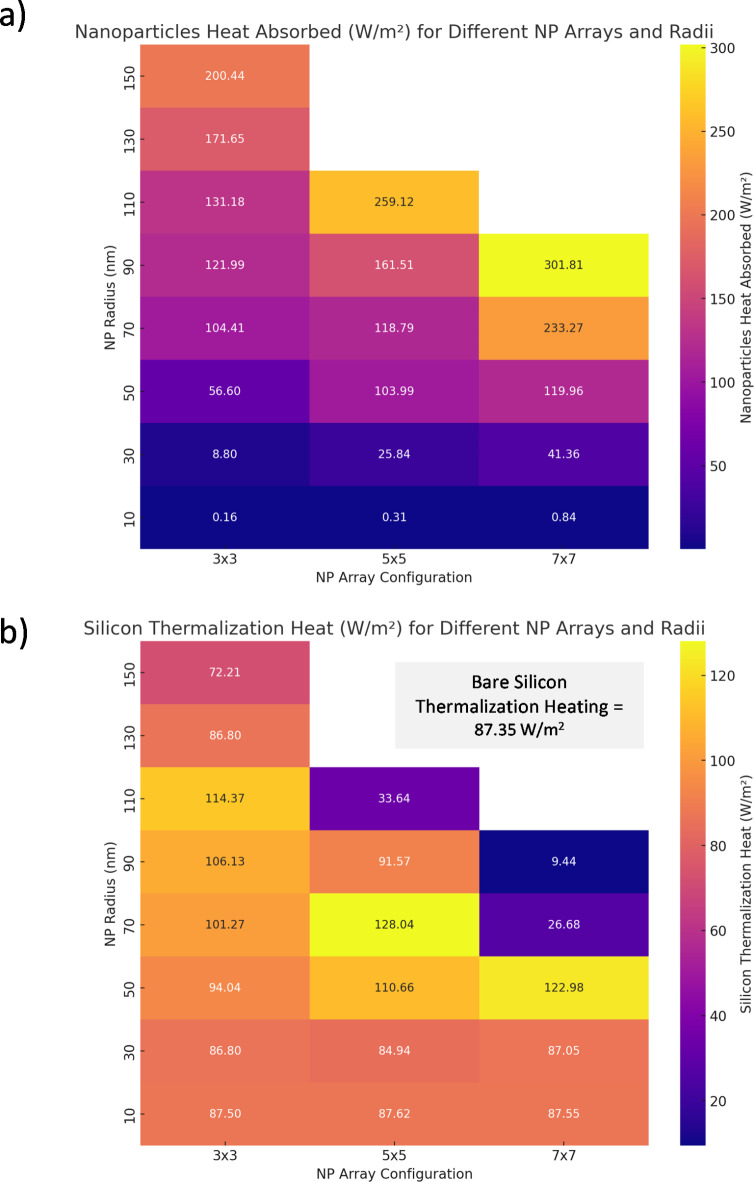
Total spectral heat absorbed divided into (**a**) Heat absorbed in NP array and (**b**) Thermalization heat content in Silicon absorber with NP configurations. Inset includes thermalization heating for bare silicon absorber for comparison. Figure created by Python 3.11.7, Dec. 2023, https://www.python.org.

Figure 8 shows the $\:{Q}_{total\:heat\:}$ separated into its constituents, namely heat absorbed in the NP array $\:{P}_{abs,NP}$ and thermalization heat content in silicon absorber $\:{P}_{therm,Si}$. The heat maps provide a detailed visualization of how different configurations and sizes of nanoparticle arrays influence heat absorption and thermalization in silicon-based systems. Figure 8a highlights the impact of NP inclusion and shows a direct relation to overall heating, which demonstrates that when the array size and radii increase, $\:{P}_{abs,NP}$ also increases. However, thermalization heat content in the silicon absorber is dependent on the maximum absorption in the carrier generation and is shown to be maximum at the optimal configuration (Fig. 8b). From Fig. 8a, it is evident that the heat absorbed by nanoparticles increases significantly with both the size of the nanoparticles and the density of the array. For instance, a 7 × 7 array with a nanoparticle radius of 150 nm shows a peak absorption of 301.81 W/m², a substantial increase compared to smaller arrays and radii. This suggests that larger and denser arrays enhance light trapping but increase local temperatures as well. In contrast, panel (b) illustrates the thermalization heat in silicon, which also varies with the array configuration and nanoparticle size. Notably, the thermalization heat does not always align proportionally with the nanoparticle absorption; for example, a 5 × 5 array with a nanoparticle radius of 90 nm exhibits a higher thermalization heat (128.04 W/m^2^) compared to some larger array configurations. This indicates that while larger nanoparticles can absorb more energy, the efficiency of converting this energy into useful thermal energy in silicon might be optimized at intermediate sizes and densities as it is also a function of the bandgap of the material.

**Fig. 9:**
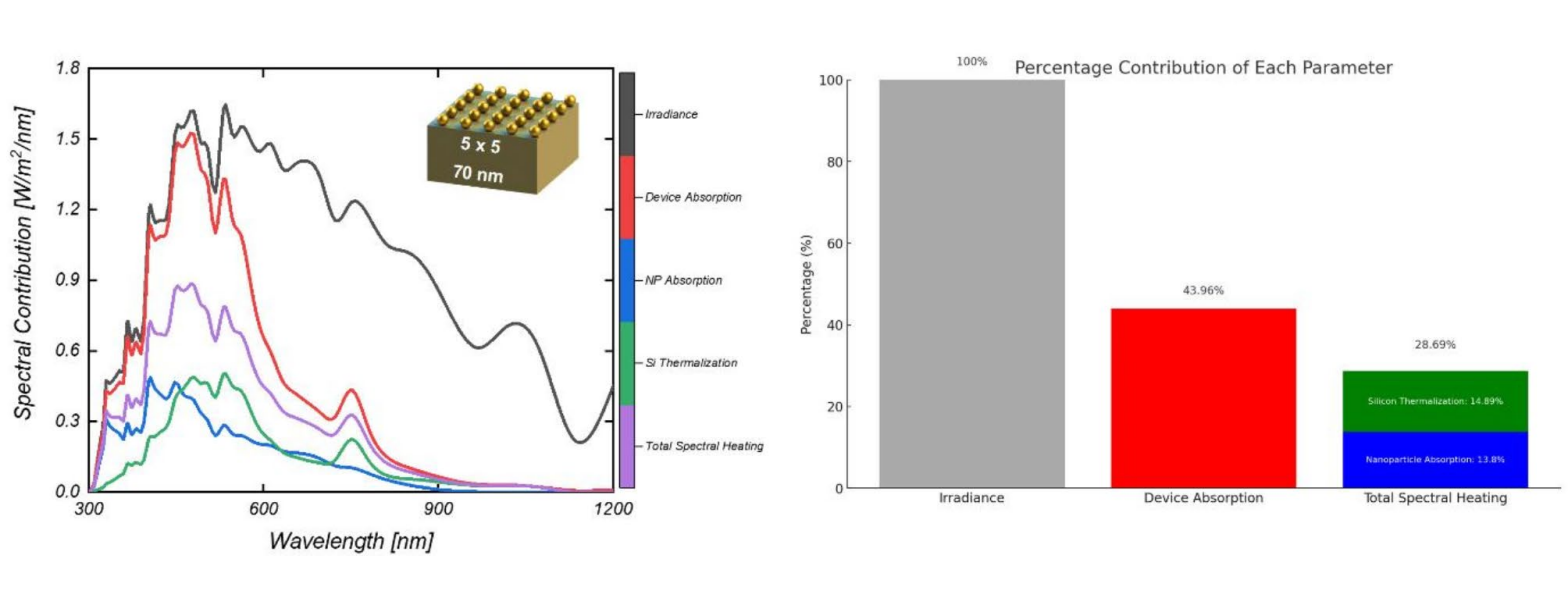
(**a**) Spectral approach to assess different thermal processes in the device for an optimal configuration (70 nm and 5×5), and (**b**) Percentage contribution of thermal processes from integrated values.

To analyze the thermal performance of the plasmonic Schottky solar cells, Fig. 9a illustrates a spectral methodology used to examine various thermal processes within the device configured optimally at 70 nm and 5 × 5 array size. This figure also quantifies the percentage contributions of these thermal processes based on integrated values. The expected spectral heating of the cells is calculated using AM1.5G solar irradiance, spectral absorption in the device, and the total spectral heat absorbed by the device (spectral heating from nanoparticles and thermalization losses in silicon absorber). This approach provides a comprehensive assessment of how thermal energy is generated and managed within the solar cell, which is crucial for optimizing device performance and longevity^42,43^. The bar graph in Fig. 9b quantifies the percentage contributions of each parameter, with Device absorption contributing the most at 43.96%, highlighting its importance in the efficiency of the solar cells. The combination of NPs absorption and thermalization ($\:{Q}_{total\:heat\:}$ (*λ*) )contributes approximately 28.69%, indicating additional energy dissipation mechanisms that impact cell performance. It can be further divided into absorption in nanoparticles (($\:{P}_{abs,NP}$ (*λ*) = 13.80%) and thermalization heating ($\:{P}_{therm,Si}$ (*λ*) = 14.89%) as shown in (Fig. 7b). This detailed breakdown aids in optimizing the plasmonic structures in solar cells by highlighting how effectively different wavelengths are converted into usable energy and identifying potential losses, guiding enhancements in plasmonic designs to boost overall cell efficiency.

**Fig. 10:**
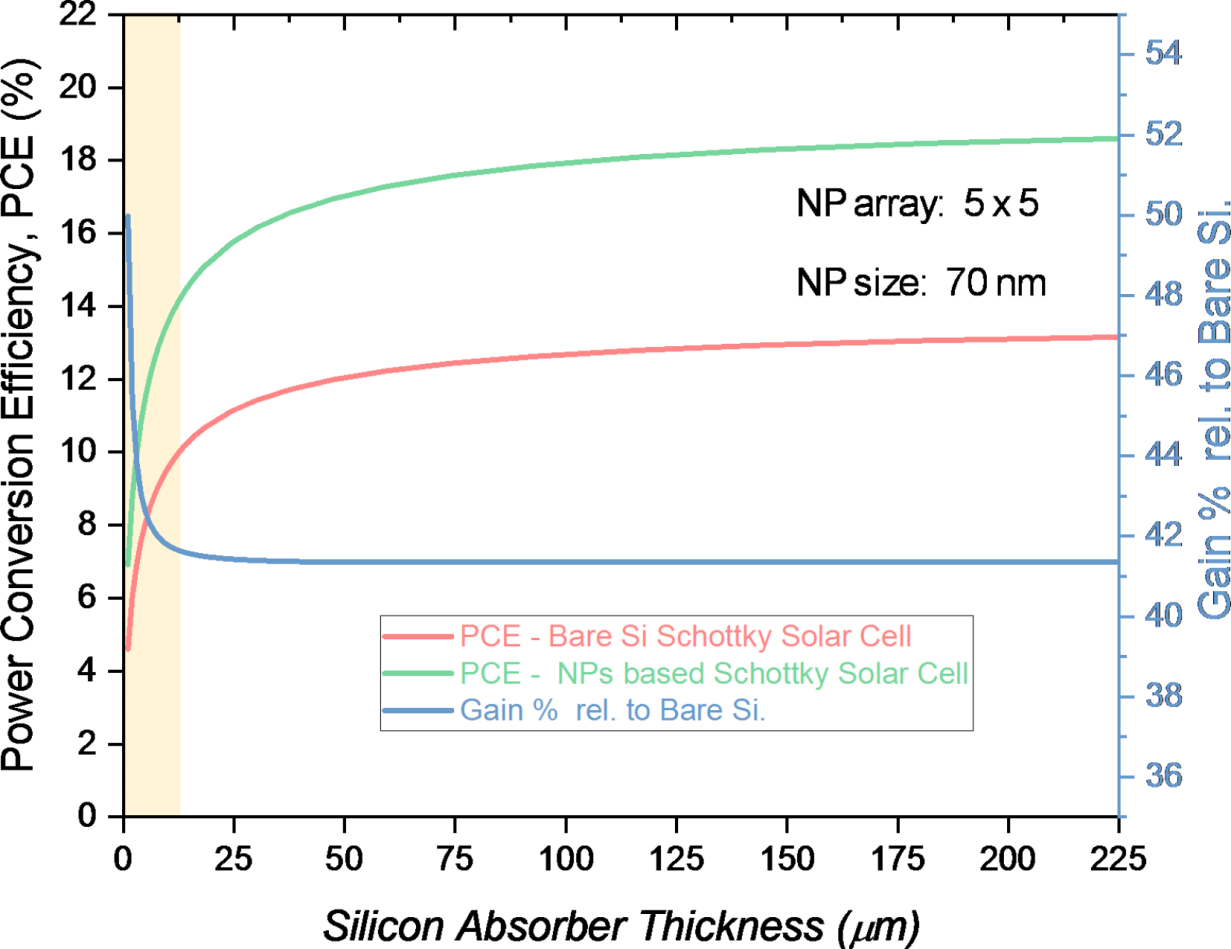
Power conversion efficiency (PCE) as a function of silicon absorber thickness for bare silicon Schottky solar cells and Schottky cells enhanced with a 5 × 5 array of 70 nm Au NPs. Gain in efficiency relative to the bare silicon cell is also plotted on right y-axis, highlighting the substantial performance saturation after few microns thickness.

Figure 10 examines the relationship between substrate thickness and Power Conversion Efficiency (PCE) in Schottky solar cells, focusing on bare silicon and silicon augmented with gold nanoparticles (Au NPs). As the substrate thickness increases from 1 μm to 5 μm, both the bare silicon and nanoparticle-enhanced solar cells exhibit a notable improvement in PCE. For bare silicon Schottky cells, PCE rises from 4.61% at 1 μm to 8.09% at 5 μm, whereas for the cells integrated with Au NPs, PCE improves from 6.91 to 11.54% over the same thickness range. Comparison between the bare silicon and nanoparticle-enhanced cells shows a consistent improvement in PCE for the Au NP-enhanced devices across all thicknesses. Gain %—which quantifies the improvement due to the inclusion of plasmonic Au NPs—starts at approximately 50% for a 1 μm thick cell but decreases to around 42.6% for a 5 μm thick cell. This trend suggests that plasmonic enhancement is more effective in thinner substrates, with the primary plasmonic effects, such as localized surface plasmon resonances (LSPRs), contributing significantly to the improvement in light absorption near the surface of the silicon. In these thin layers, the LSPRs in Au NPs lead to enhanced near-field scattering and localized electric field intensification, which directly increase the absorption of incident photons within the silicon substrate^42^.

As the substrate thickness increases, the relative contribution of the plasmonic effects diminishes because the bulk silicon begins to dominate the absorption process. In thicker substrates, the volume available for photon absorption increases, but the enhancement provided by the plasmonic scattering from Au NPs becomes less pronounced relative to the overall absorption in the bulk. This is consistent with the nature of surface plasmon resonance, where the light-matter interaction is highly localized within the near-field region of the nanoparticles, typically within the first few hundred nanometers to a few microns from the surface^12,24^. As a result, increasing the thickness beyond the first few microns primarily improves bulk absorption but has less impact on enhancing light trapping through plasmonic scattering^43^. Moreover in Schottky solar cells, the key physical processes—such as carrier generation, separation, and collection—are localized near the metal-semiconductor interface and within the depletion region, which is generally confined to the first few microns of the substrate. Therefore, thinner cells are sufficient to capture the essential physics of these devices^43^. Specifically, in the presence of Au NPs, the plasmonic resonances significantly boost the optical path length of the incident light through scattering and near-field coupling, which increases the absorption cross-section of the silicon layer. This effect is most pronounced within a thin silicon layer, where the enhanced scattering can effectively trap light in the active region, leading to improved PCE. Additionally, thicker substrates reduce the efficiency of carrier collection, as charge carriers generated deep within the substrate are more likely to recombine before reaching the Schottky barrier, thus lowering the overall device efficiency. Therefore, while thicker cells improve photon absorption, they do not capture the key advantages of plasmon-enhanced scattering as effectively as thinner cells^42,43^.

From a design perspective, the results indicated that thinner Schottky solar cells, particularly in the 2–3 μm range, can efficiently capture the critical physics of plasmonic enhancement. In this regime, the localized surface plasmons in Au NPs lead to significant improvements in light trapping and absorption, optimizing the use of the substrate material and achieving high PCE without the need for excessively thick substrates.

**Fig. 11:**
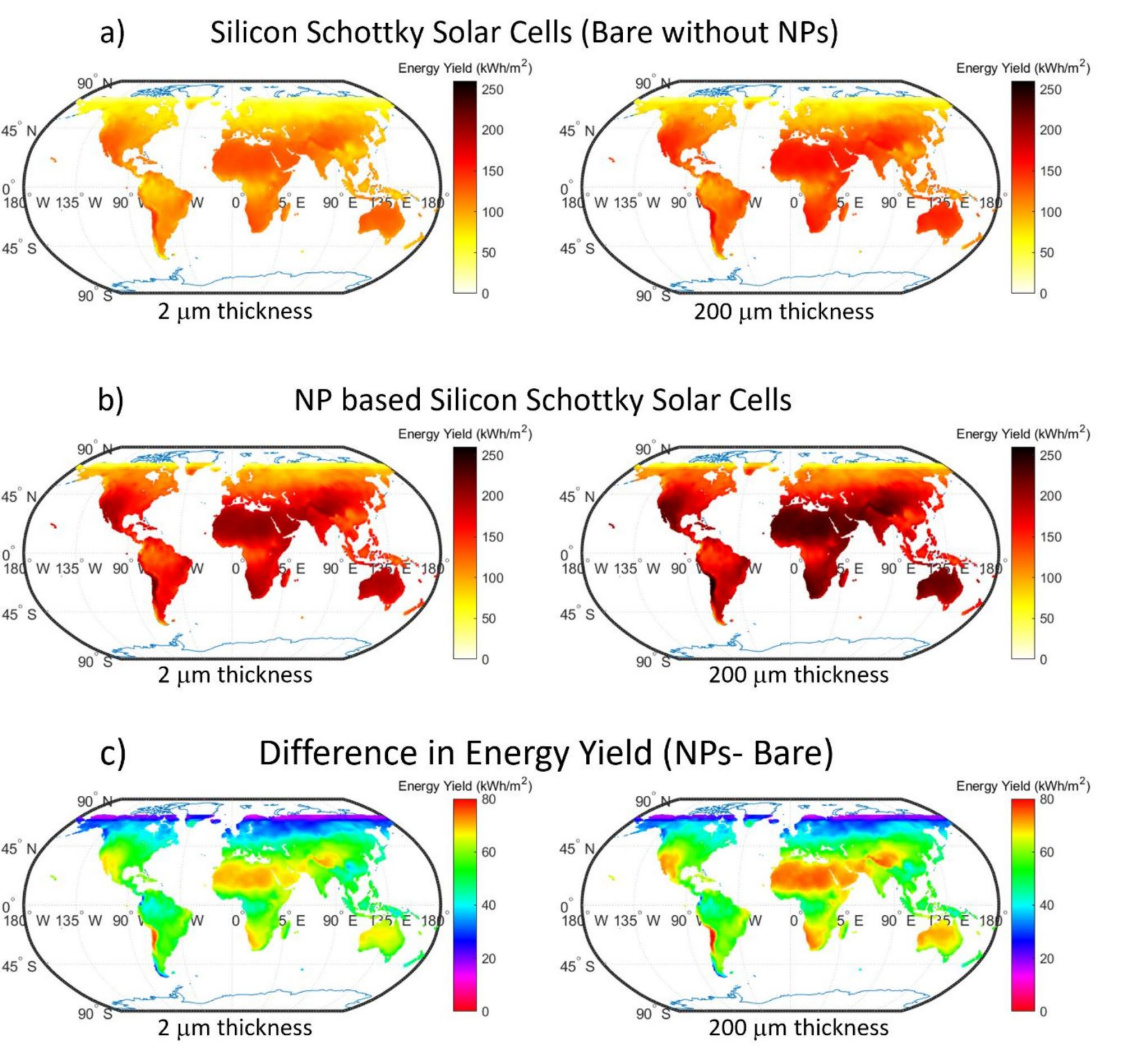
Global maps of energy yield for silicon Schottky solar cells, comparing cells (**a**) without nanoparticles and (**b**) with nanoparticles optimal PSSC (5×5, 70 nm), and (**c**) highlighting the yield differences with bare-Si Schottky solar cell. Figure created by MATLAB 2019a, https://www.mathworks.com/products/matlab.html.

Figure 11 shows a global potential map of the annual energy difference between optimal PSSC (5 × 5, 70 nm) device and bare-Si Schottky Solar cell. It provides a detailed comparison of energy yields for Schottky silicon solar cells with and without plasmonic nanoparticle (NP) enhancements across two silicon absorber thicknesses: 2 and 200 *µm*. The energy yield (EY) values are analyzed across global locations based on latitude and longitude while taking into account factors such as annual plane of array (POA) radiation and ambient temperature.

Bare silicon cells without nanoparticles show varied energy outputs globally, predominantly higher in regions with strong sunlight (Fig. 11, panel a). However, with the inclusion of metallic nanoparticles, these cells exhibit significantly elevated energy yields across all geographic areas (Fig. 11, panel b). Through the stimulation of plasmonic gold nanoparticles, light enhancement in these devices takes place. For the thinner 2 μm silicon Schottky solar cell (left column), the mean energy yield (EY) for the bare design (without nanoparticles) is approximately 95.82 kWh/m². The plasmonic NP-enhanced design, however, demonstrates a notable increase in energy yield, with a global mean of 145.37 kWh/m². The mean energy yield difference between the NP-enhanced and bare designs is approximately 49.55 kWh/m², indicating a significant improvement due to the plasmonic enhancement. This increase can be attributed to the enhanced light absorption provided by the plasmonic nanoparticles, which are particularly effective in boosting energy conversion efficiency for thinner silicon absorbers(Fig. 11, panel c). The consistent improvement across a wide geographic range suggests that the light-trapping mechanisms induced by the nanoparticles are highly effective in optimizing absorption even when the absorber thickness is limited. In comparison, current state-of-the-art silicon absorbers usually employ 150–200 *µm* thick absorbers^43–47^.

For the thicker 200 μm silicon Schottky solar cells, the mean energy yield for the bare design is higher, at 112.78 kWh/m^2^, with a standard deviation of 31.17 kWh/m^2^. The plasmonic NP-enhanced design again shows a significant enhancement, with a mean energy yield of 165.14 kWh/m^2^ and a standard deviation of 46.08 kWh/m^2^. The mean energy yield difference between the NP-enhanced and bare designs is 52.36 kWh/m^2^. The energy yield enhancement in the 200 μm design, while slightly higher than in the 2 μm case, is less pronounced as a relative percentage, indicating that while plasmonic nanoparticles still provide substantial benefits, the thicker silicon absorber already possesses inherent light absorption capacity. However, the plasmonic enhancement remains beneficial, particularly in the short-wavelength range where the bare silicon’s absorption may be less efficient.

While the ambient temperature can have a modest impact on the absolute energy yield, the relative improvement due to the plasmonic nanoparticles appears to be largely unaffected by temperature variations. This robustness indicates that plasmonic NP-based solar cells can maintain high performance across a diverse range of climatic conditions, making them highly suitable for global deployment.

## Conclusions

This paper explores the development of an opto-thermal-electrical model for plasmonic Schottky solar cells (PSSC) using a comprehensive multiphysics approach. By employing tools such as COMSOL, MATLAB, and SCAPS, we simulated the optical properties and energy conversion efficiencies of PSSCs with varying nanoparticle (NP) configurations and sizes. By enhancing optical absorption for improved carrier generation, we accounted for thermal effects that may reduce efficiency, particularly in hot climates. This comprehensive approach allows us to effectively evaluate the overall efficiency and annual energy yield of the solar cells. Our spectral analysis focused on the absorption characteristics of these solar cells, as indicated in the absorption spectrum for systems sized 3 × 3, 5 × 5, and 7 × 7, with NP radii ranging from 10 nm to 150 nm. The simulations demonstrated that a 5 × 5 NP array with a 70 nm radius provided optimal electrical performance, achieving a short circuit current (Jsc) of 11.54 mA/cm^2^, which represents a 46.8% gain over a bare silicon (Si) Schottky solar cell. This configuration yielded an optimal fractional coverage of 34.9%, indicating an efficient use of the frontal area of NPs atop the Si absorber. The thermal analysis highlighted the various thermal processes within the device, showing that device absorption contributed the most at 43.96% to the overall energy handling, with additional significant contributions from NP absorption and thermalization, indicating complex energy dissipation mechanisms within the cell. Notably, the optimal configuration of a 5 × 5 array with a 70 nm NP diameter resulted in a total spectral heat absorption of 246.83 W/m², demonstrating less heating compared to configurations with larger or denser NP arrays. This study guides the optimization of plasmonic structures, aiming to boost the overall efficiency of Schottky solar cells. Thinner 2 μm absorber benefits more from the light-trapping effects of the nanoparticles, but even in thicker 200 μm designs, the plasmonic enhancement provides a considerable boost in performance. These findings highlight the versatility and effectiveness of plasmonic designs in improving solar cell efficiency across different absorber thicknesses and environmental conditions. The mean improvements of 49.55 kWh/m² for the 2 μm cells and 52.36 kWh/m² for the 200 μm cells underscore the potential of this technology to enhance solar energy yield in a wide range of applications.

## Data Availability

The datasets used and/or analyzed during the current study available from the corresponding author on reasonable request.
